# Comparison of the timed inspiratory effort index with the T-piece trial as a decision-making tool for extubation: a randomized controlled non-inferiority trial

**DOI:** 10.1590/1414-431X2023e12824

**Published:** 2023-08-14

**Authors:** M.D.P. Godoy, L.C. de Souza, A.E. da Silva, J.R. Lugon

**Affiliations:** 1 Programa de Pós-graduação em Ciências Médicas, Faculdade de Medicina, Universidade Federal Fluminense, Niterói, RJ, Brasil; 2 Serviço de Fisioterapia, Hospital Icaraí, Faculdade de Fisioterapia, Universidade Estácio de Sá, Niterói, RJ, Brasil; 3 Hospital Icaraí, Fisioterapia, Niterói, RJ, Brasil; 4 Departamento de Medicina/Nefrologia, Faculdade de Medicina, Universidade Federal Fluminense, Niterói, RJ, Brasil

**Keywords:** Mechanical ventilation, Respiratory failure, Weaning ventilation, Weaning predictive index, Spontaneous breathing trial, Respiratory muscle

## Abstract

The “timed inspiratory effort” (TIE) index, a new predictor of weaning outcome, normalizes the maximal inspiratory pressure with the time required to reach this value up to 60 s, incorporating the time domain into the assessment of inspiratory muscle function. The objective of this study was to determine whether the TIE predicts successful extubation at a similar rate as the T-piece trial with less time required. A non-inferiority randomized controlled trial was performed with ICU subjects eligible for weaning. The participants were allocated to the TIE or the T-piece groups. The primary outcome was successful weaning, and the main secondary outcome was ICU mortality. Eighty participants of each group were included in the final analysis. Time from the start of a successful test to effective extubation was significantly lower in the TIE group than in the T-piece group, 15 (10 to 24) *vs* 55 (40 to 75) min, P<0.001. In the Kaplan-Meier analysis, no significant differences were found in successful weaning (79.5 *vs* 82.5%, P=0.268) or survival rate (62.9 *vs* 53.8%, P=0.210) between the TIE and T-piece groups at the 30th day. In this preliminary study, the TIE index was not inferior to the T-piece trial as a decision-making tool for extubation and allowed a reduction in the decision time.

## Introduction

In the intensive care environment, mechanical ventilation is an essential technique for life maintenance. However, it can be associated with several complications and increased costs related to the procedure's duration (1). In some reports, weaning accounts for more than 40% of the total time of mechanical ventilation (2-6).

A recently described weaning predictor, the “timed inspiratory effort” (TIE) index, normalizes maximal inspiratory pressure (PImax) by time required to reach this value, thus incorporating the time domain into the assessment of inspiratory muscle function (7,8). It is calculated as the ratio of PImax (after the first 30 s of observation) by time required to reach the PImax, under occlusion of the airways by a unidirectional exhalation valve for up to 60 s. Values of TIE of 1.0 cm H_2_O/s or higher have been associated with a very high probability of successful weaning in previous studies of our group, performing better than the PImax (7-10).

One issue to consider about the weaning and extubation process is the time it takes to decide whether to extubate (4, 11-14). We wondered if it is worth waiting 30 min using the T-piece strategy if the decision to extubate can be made within one minute or less. It should be emphasized that, to our knowledge, no study has compared the performance of a weaning predictor with a spontaneous breath trial as a decision-making tool for extubation. We hypothesized that the TIE index predicts successful extubation at a similar rate as the spontaneous breath trial with less time required.

## Material and Methods

### Design and settings

This was a prospective non-inferiority randomized controlled trial (RCT) enrolling subjects mechanically ventilated admitted to the intensive care unit (ICU) of a hospital located in the state of Rio de Janeiro, Brazil, who were in the process of weaning. The tools used for extubation decision-making were the TIE index or the traditional T-piece trial (TPT). Before starting data collection, we used an online number generator (https://www.random.org/sequences/) to produce a random sequence of numbers from 1 to 160 in a two-column format (TIE or TPT groups). Each participant received a number according to the order of entry into the study. The column to which the assigned number belonged was used to allocate the patient to one of the two groups. The study was approved under the number 1.917.979 by the Research Ethics Committee of the Medical School of Universidade Federal Fluminense in February 2017, but effective enrollment was only started on October 29, 2019, finishing on October 17, 2022. The study was registered at “ClinicalTrials.gov” (ID: NCT 04512677) on August 12, 2020. An informed consent was obtained from each subject or their next of kin before enrollment.

### Inclusion and exclusion criteria

Our study included subjects over 18 years old, under mechanical ventilation, who remained for more than 24 h on invasive ventilatory assistance and were able to start the process of weaning. Participants should be alert (defined by the ability to perform objective actions, such as opening the eyes in response to a voice or a RASS score of -1 to +1), have overcome the acute phase of their disease, have a cough reflex, and have no excessive tracheobronchial secretion. Infection should be under control, cardiovascular status stable (heart rate ≤120 beats/min, and systolic blood pressure between 90 to 160 mmHg, without or on a low-dose of vasopressor), hemoglobin ≥8 g/dL, arterial oxygen saturation >90% with an inspired fraction of oxygen (FIO_2_) ≤0.4 or the ratio of the partial pressure of oxygen in arterial blood by the inspiratory fraction of oxygen ≥200 with final positive expiratory pressure ≤8 cm H_2_O, respiratory rate ≤35 breaths/min, supportive pressure ≤10 cmH_2_O, pH >7.30, and a temperature <38°C (5-7,12,15).

Exclusion criteria were tracheostomy, chronic neurological disorders, low level of alertness, severely reduced left ventricular ejection fraction, and positive serology for HIV.

### General procedures

The TIE index incorporates the time domain into the assessment of muscle inspiratory function. It is calculated as the ratio of PImax registered after the first 30 s of observation by the time required to reach it, while keeping the airways occluded with a unidirectional valve for up to 60 s. The TIE Meter^®^ digital vacuometer (Magnamed, Brazil; Supplementary Video S1), with a 300 cmH_2_O scale of 0.1 cmH_2_O increments and time intervals of 0.01 s between each pressure measurement was used to measure the PImax and the TIE index.

Before testing, all participants were ventilated under pressure-support mode (≤10 cmH_2_O). Subjects were positioned in dorsal decubitus with the head elevated at 45°, and the cuff was inflated to prevent leakage during measurement. After tracheal aspiration, the subjects remained connected to the mechanical ventilator with a 100% fraction of inspired oxygen (FIO_2_) for two minutes to prevent hypoxemia during measurements (7,8,15,16). Then, the mechanical ventilator was disconnected, and the digital vacuometer was coupled to the orotracheal tube. The inspiratory pressure values in cmH_2_O and their corresponding time-points in seconds were stored and analyzed without verbal commands. Measurements were taken under strict monitoring and values of the TIE index ≥1 cmH_2_O/s (cutoff) were required for a favorable decision to extubate (7-10,15,16).

For the spontaneous breathing trial, subjects were positioned in dorsal decubitus with the head elevated at 45° with a low-flow oxygen support (∼40% FIO_2_). They were connected to a T-piece for 30 min and monitored for possible signs of intolerance such as respiratory rate >35 breaths/min, arterial oxygen saturation <90%, heart rate >120 beats/min, systolic blood pressure >180 or <90 mmHg, and signs and symptoms of agitation, diaphoresis, or alteration of alertness. The subjects who kept their respiratory pattern, had blood gas analysis and hemodynamic stability, and seemed comfortable for the 30 min were considered successful in the T-piece trial (3,6,17,18).

The criteria to proceed with extubation was either a TIE index ≥1 cm H_2_O/sec or a successful T-piece trial. A 24-h time interval was required for reassessment of participants who did not complete the TIE measurement due to adverse events or had a value <1.0 cm H_2_O/s; the same interval was adopted for those who failed the T-piece trial. Weaning was deemed successful if the patients remained on spontaneous ventilation in the ICU for at least 48 h following extubation, without the need to receive non-invasive ventilatory support (CPAP/BiPAP) or a high-flow nasal cannula (3,4,17,18). For the study, weaning failure was defined as reintubation before 48 h with return to mechanical ventilation.

### Outcomes

The primary outcome of the present study was successful weaning. Following the consensus definition that prevailed at the time of the study design, it was defined as sustained spontaneous breathing for >48 h in the ICU after withdrawal from mechanical ventilation (MV) (6,18).

The secondary outcomes were: ICU mortality, duration of weaning process, time from the decision to perform the test to extubation, frequency of extubation after the first, second, and third tests, frequency of reintubation before 48 h, frequency of reintubation after 48 h, frequency of tracheostomies after reintubation, and length of stay in the ICU (until discharge or death) from ICU admission.

### Statistical analysis

We calculated that a sample of 80 subjects per group would provide the trial with 80% power to detect non-inferiority of the primary outcome at an alpha of 0.05 and a non-inferiority margin of 15 percentage points, assuming a dropout rate of 10%, and that ∼80% of the subjects in the TIE index group and the T-piece trial group would have successful weaning (19,20).

The Kolmogorov-Smirnov test was used to assess the distribution pattern of the variables. Results are reported as mean and standard deviation for normal distribution or median and interquartile range otherwise. Differences between continuous variables were compared with the two-tailed *t*-test for Gaussian distribution or with the Mann-Whitney test, alternatively. Categorical variables are reported as frequencies, and differences were compared with the chi-squared or Fisher exact test. Kaplan-Meier curves with log-rank tests were used to compare time for successful weaning or death rates in the study groups. Statistical significance was set at P<0.05. Statistical analyses were performed using SPSS software version 18.0 for Windows (IBM, USA).

## Results

**Figure 1:**
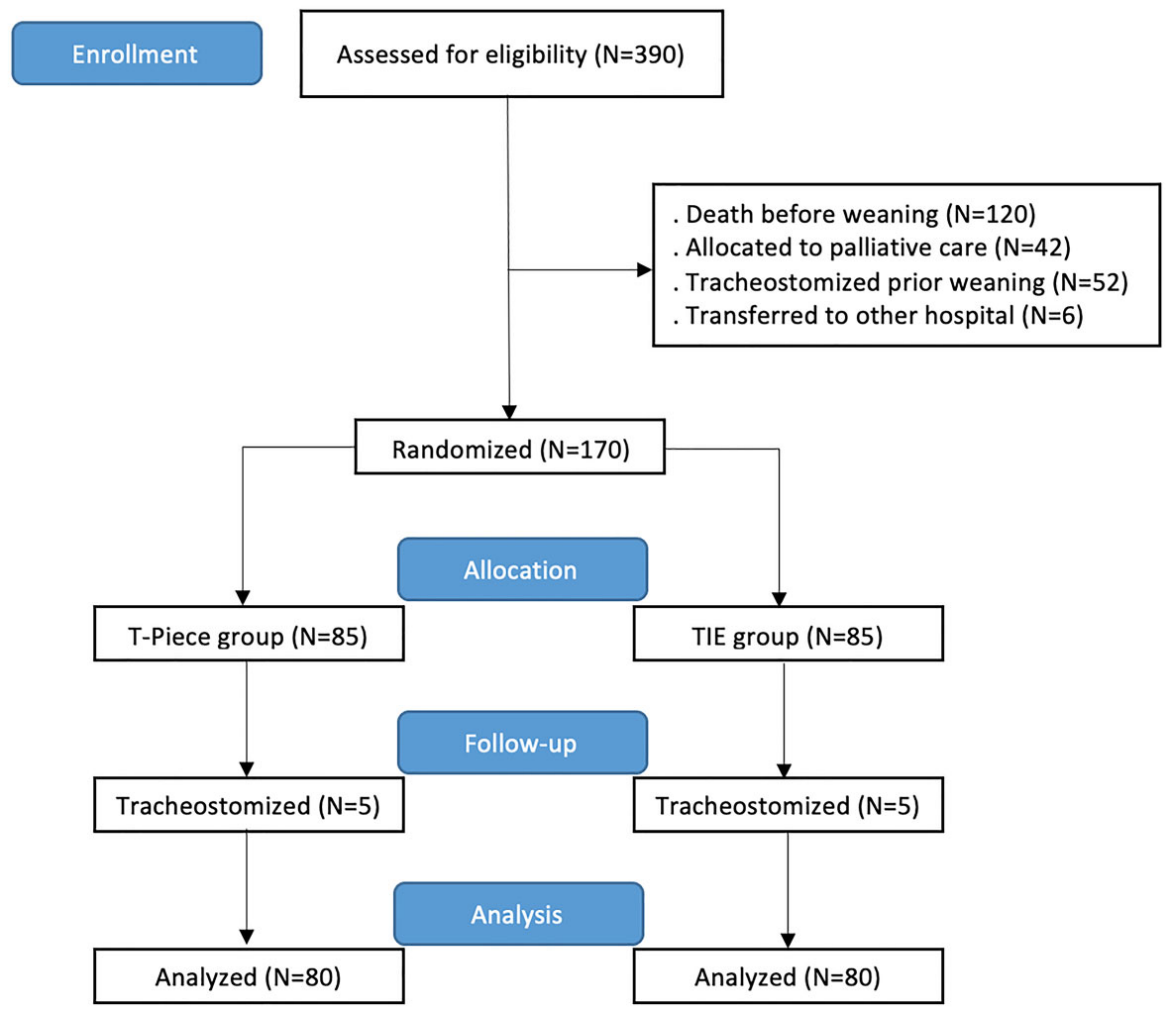
Flow chart of participants of the intervention and control groups. TIE: timed inspiratory effort.

The study analyzed a sample of patients recruited from October 2019 to October 2022. Three hundred and ninety individuals were mechanically ventilated and assessed for eligibility. After applying the exclusion criteria, 170 were found to be apt for randomization. Before the first weaning attempt, five subjects in each study arm underwent tracheostomy and were never extubated. The final analysis was performed with 80 participants in each group (Figure 1).

The general characteristics of the 160 participants included in the study analysis are shown in Table 1. The randomization resulted in male gender preponderance in the T-piece group (53.8 *vs* 36.3%).

**Table 1:** General characteristics of participants at baseline.

Variables	TIE (n=80)	TPT (n=80)
Male gender, n (%)	29 (36.3)	43 (53.8)
Age, years	75 (64-81)	69 (58-82)
APACHE II score	17 (14-22)	19 (17-20)
Prior use of glucocorticoid, n (%)	19 (23.8)	14 (17.5)
Prior use of neuromuscular blocker, n (%)	15 (18.8)	14 (17.5)
Renal replacement therapy, n (%)	19 (23.8)	16 (20.0)
Time of MV until first weaning attempt, days	5 (3-8)	5 (3-7)
Conditions precipitating ICU admission, n (%)		
Sepsis	30 (37.5)	32 (40.0)
COVID-19	11 (13.8)	13 (16.3)
Stroke	15 (18.8)	5 (6.3)
Heart failure	7 (8.8)	11 (13.8)
Surgery conditions	3 (3.8)	11 (13.8)
COPD	5 (6.2)	2 (2.5)
Miscellaneous	10 (12.5)	6 (7.5)

Data are reported as number and percent or median (interquartile range). TIE: timed inspiratory effort; TPT: T-piece trial; APACHE II: Acute Physiology and Chronic Health Evaluation II; MV: mechanical ventilation; ICU: intensive care unit; COVID-19: SARS-CoV-2 disease; COPD: chronic obstructive pulmonary disease.

Data referring to clinical and laboratory parameters on the day of the first decision test and test-related adverse events are shown in Table 2. Values of C-reactive protein were higher in the T-piece group: 4.1 (1.8 to 5.9) *vs* 2.7 (1.1 to 3.3) mg/dL, P<0.001. In addition, serum bicarbonate values were significantly higher in this group, 26 (25 to 31) *vs* 25 (23 to 27) mmol/L, P=0.009, as well as the values of PCO_2_, 42.3 (37 to 46) *vs* 39.3 (36 to 45) mmHg, P=0.034, and SaO_2_, 98 (98 to 99) *vs* 98 (97 to 99) %, P=0.020.

**Table 2:** Laboratory results at the day of the first test and baseline clinical parameters in each group.

Variables	TIE (n=80)	TPT (n=80)	P-value
Laboratory variables at testing day			
Leukocytes/mm^3^×10^3^	9.8 (7.7-12.4)	10.3 (7.9-12.5)	0.524
Hemoglobin, g/dL	10 (9-12)	10 (9-11)	0.284
C-Reactive protein, mg/dL	2.7 (1.1-3.3)	4.1 (1.8-5.9)	<0.001
pH	7.40 (7.39-7.47)	7.41 (7.39-7.50)	0.610
PO_2_, mmHg	95 (81-116)	102 (86-126)	0.085
PCO_2_, mmHg	39.3 (36-45)	42.3 (37-46)	0.034
HCO_3_, mmol/L	25 (23-27)	26 (25-31)	0.009
SaO_2_, %	98 (97-99)	98 (98-99)	0.020
Clinical variables before the test			
Heart rate, beats/min	90 (81-98)	90 (77-99)	0.548
Respiratory rate, breaths/min	21 (19-23)	20 (18-22)	0.020
Systolic blood pressure, mmHg	132 (113-147)	136 (120-148)	0.238
FIO_2_, %	30 (28-32)	30 (28-30)	0.691

Data are reported as median (interquartile range). Mann-Whitney test was used to compare medians. TIE: timed inspiratory effort; TPT: T-piece trial.

In the TIE group, 7 (10%) patients did not complete the test due to adverse events. Heart rate >120 beats/min and respiratory rate >35 breaths/min were present in 7 (10%) of the cases, arrhythmia in 1 (1%), mean blood pressure <70mmHg in 2 (3%), and SpO_2_ <90% in 5 (7%). Twelve patients (18%) failed the T-piece test. Heart rate >120 beats/min was found in 11 (16%) cases, respiratory rate >35 breaths/min in 12 (18%), mean blood pressure <70 mmHg in 4 (6%), and SpO_2_ <90% in 5 (7%).

The metrics of the mechanical ventilation process are shown in Table 3. A statistically significant difference was found in the time from the start of a successful test to effective extubation, which was lower in the TIE group, 15 (10 to 24) *vs* 55 (40 to 75) min, P<0.001 (data not shown in the table).

**Table 3:** Metrics of the mechanical ventilation process in the study groups.

Variables	TIE (n=80)	TPT (n=80)	P-value
Days of MV until the first weaning attempt	5 (3-8)	5 (3-7)	0.051
Length of weaning process, days	2 (0-3)	1 (1-3)	0.735
Results favoring the decision of extubation in the first test, n (%)	73 (91)	72 (91)	0.99
Results favoring the decision of extubation in the 2nd and 3rd tests, n (%)	6 (7)/1 (1)	8 (10)/0 (0)	0.467
Reintubation before 48 h, n (%)	10 (12.5)	15 (18)	0.276
Reintubation after 48 h, n (%)	8 (10)	4 (5)	0.230
Tracheostomies after reintubation, n (%)	5 (6)	8 (10)	0.385
Length of stay in ICU, days	20 (13-24)	17 (12-22)	0.32

Data are reported as median (interquartile range), unless otherwise indicated. Mann-Whitney or chi-squared test. TIE: timed inspiratory effort; TPT: T-piece trial; MV: mechanical ventilation; ICU: intensive care unit.

**Figure 2:**
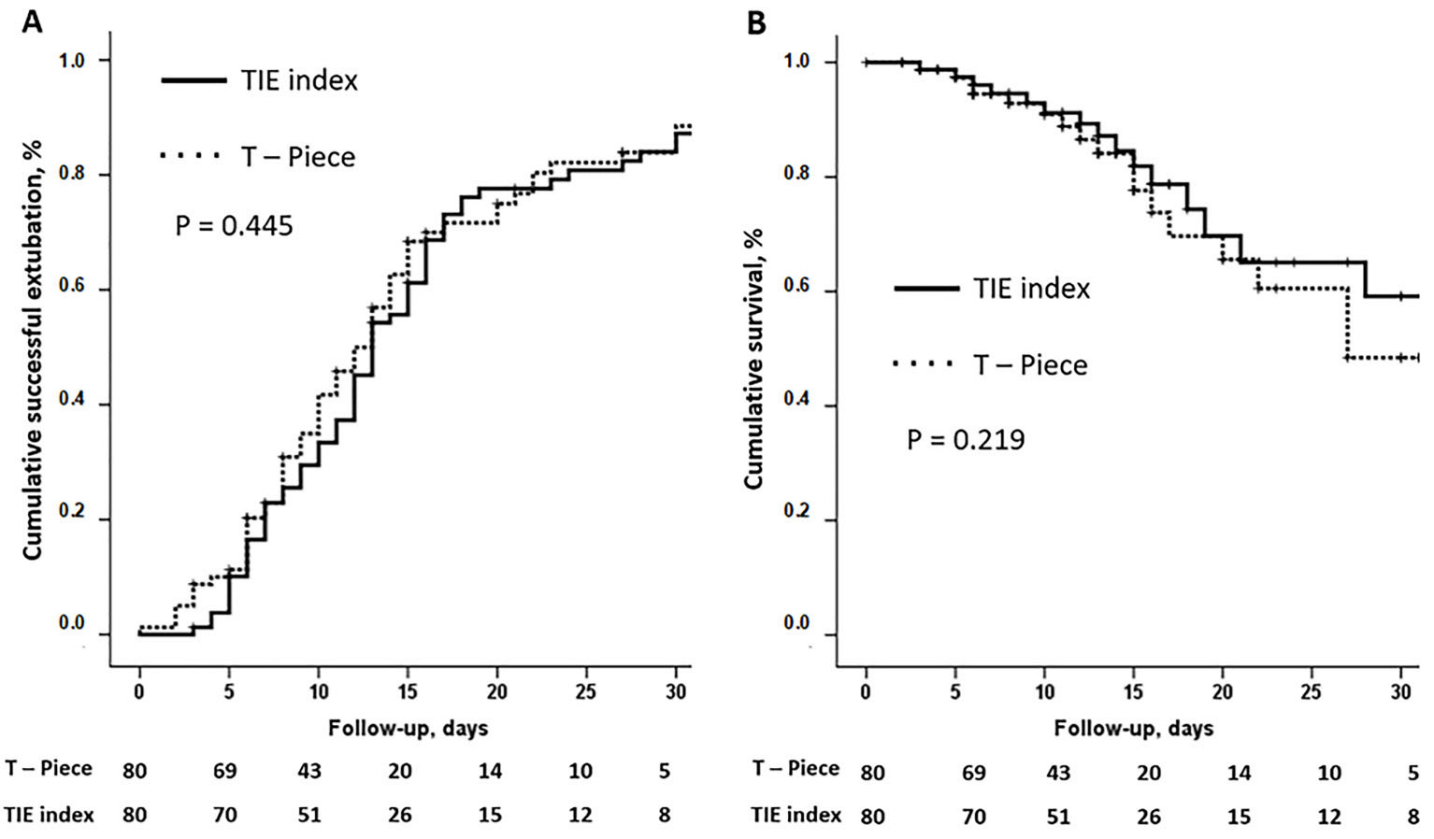
Kaplan-Meier curves showing the cumulative successful weaning (**A**) and cumulative survival (**B**) in the first 30 days in the intensive care unit in each group. The follow-up period was counted from the start of the weaning process. Events in A correspond to the date of the extubation.

The cumulative survival rate and the cumulative successful weaning rate in each group are shown in Figure 2. There was no significant difference between the groups on the 30th day regarding successful weaning rate (82.4 *vs* 83.9%, P=0.445) or survival rate (65.1 *vs* 54.5%, P=0.219) in TIE and T-piece groups, respectively.

## Discussion

The extubation decision-making process has been carried out based on the result of a spontaneous breathing test, irrespective of the results of the predictive indices (3,6,21-26). In the present study, we chose to use the TIE index (taking into consideration its higher predictive value - AUC >0.90) (7-10,15,16) as an extubation decision tool and compared its performance with that of the traditional T-piece spontaneous breathing trial. Given the impossibility of using a placebo group, the non-inferiority trial was chosen to compare this new strategy that completed in one or fewer minutes with the standard intervention that takes 30 min or more to perform (20).

The decision to use the T-piece, the strategy usually employed in our hospital, instead of the pressure support ventilation (PSV) was made based on observations suggesting that the T-piece simulates a similar ventilatory load as after extubation and therefore assesses the subject's ability to maintain spontaneous breathing under more realistic conditions (13,27-30). Indeed, the last meta-analysis and a recent report addressing such issue did not report relevant differences in the outcomes of the two strategies (31,32). However, Subirá et al. (12) found significantly higher rates of successful extubation among patients who underwent spontaneous breathing tests in ventilation with pressure support for 30 min compared to those in T-piece ventilation for two hours.

The time to collect the data took longer than initially programmed. Like everyone else in the world, we were caught by surprise by the outbreak of the COVID-19 pandemic, which caused a temporary interruption of the study and delayed reaching the initially planned sample. It took the research team six months to become familiar with the management of the COVID-19 patients and to take precautions to prevent contamination during data collection.

Consistent with previous studies (33,34), sepsis was the most frequent diagnosis in both groups. Since part of data collection was accomplished during the pandemic, COVID-19 cases accounted for a substantial proportion of the participants. Laboratory findings at the time of performing the test to proceed with the first extubation process of the two groups were compared. The small but statistically significant differences found in serum C-reactive protein and bicarbonate levels were not clinically relevant, with the values in both groups remaining within the reference range (33,34).

In our 12-year experience using the TIE index, adverse events occurred in approximately 5 to 7% of the procedures, none of which were serious (7-10,15,16). In the present study, the frequency of adverse events that led to test interruption was low and without statistically significant differences between groups. As in our previous studies, the measurement of the TIE index performed up to 60 s after unidirectional airway occlusion was consistently safe.

As expected, the time elapsed from the beginning of the decision test until extubation was higher in the T-piece group because of the different duration of the tests. The study design accounts for such a statistically significant difference since the TIE index measurement takes about 60 s, whereas the T-piece trial, around 30 min (3,7,8). Many studies point out that prolonged mechanical ventilation time is directly related to a higher probability of extubation failure and increased mortality (17,23,34,35). The shorter median period of 48 min in favor of the TIE group may not impact patient outcome but could represent a clinical advantage by speeding extubation decision.

No difference was found regarding survival rate and successful weaning rate between the groups in the Kaplan Meier analysis. However, the speed of the extubation process resulting from using the TIE index as a decision-making tool effectively reduced the time the patient was subjected to the stress of the tests. Noticeably, the performance of the TIE index as a decision-making tool for extubation was not inferior to that of the T-piece trial for any of the study's main outcomes, weaning success and mortality.

The study had some limitations. Our local Ethics Committee approved the study in February 2017, but due to internal problems, the start of recruitment was delayed. Effective enrollment was started on October 29, 2019 and finished on February 15, 2021. The data were unblinded on March 16 2021, when the analysis was completed and we started writing the manuscript. The use of the T-piece trial could be seen as a limitation, but a recent study showed that weaning outcome when using such a strategy or pressure-support ventilation did not differ (32). Finally, our sample was relatively small and from a non-public hospital, which limits the generalizability of our findings. Nevertheless, these limitations do not detract from the valuable information provided by our RTC.

In conclusion, this preliminary study showed that the use of the TIE index as a decision-making tool for extubation was not inferior to a T-piece trial and allowed reducing the time required to make a decision. We believe that a large multicenter randomized controlled trial is needed to substantiate our findings.

## Supplementary Material

Click here to view [mp4].
